# Sexual desire for non-normative sexual behaviors: differences between centennials and millennials considering sexual orientation

**DOI:** 10.3389/fsoc.2024.1509111

**Published:** 2024-12-19

**Authors:** Alberto Paramio, Ricardo Tejeiro, Antonio Romero-Moreno, María Rusillo-Molina, Serafín Cruces-Montes

**Affiliations:** ^1^Department of Psychology, Faculty of Education Sciences, University of Cádiz, Puerto Real, Spain; ^2^INDESS (University Research Institute for Sustainable Social Development), University of Cádiz, Jerez de la Frontera, Spain; ^3^Department of Psychology, Liverpool John Moores University, Liverpool, United Kingdom

**Keywords:** sexual behavior, sexual desire, non-normative, millennials, centennials

## Abstract

**Introduction:**

Non-normative sexual behaviors were traditionally studied from a psychopathological perspective, although nowadays a distinction is made between paraphilia (nonpathological) and paraphilic disorder (mental disorder).

**Methods:**

The present study aims to examine the differences between a group of millennials (*n* = 173) and centennials (*n* = 159) in their appetite for these sexual behaviors without the preconception of these behaviors as harmful or pathological.

**Results:**

Differences in appetite related to exhibitionism and foot fetishism were found in the first instance, with millennials showing a greater appetite for these. When including sexual orientation in the analysis, in addition to exhibitionism and foot fetishism, differences were found in behaviors related to asphyxiation, bestiality and urophilia. Millennials and homobisexual centennials showed the highest appetite for exhibitionism behaviors, homo-bisexual centennials for choking behaviors and bestiality and homo-bisexual millennials for foot fetishism and urophilia-related behaviors.

**Discussion:**

Exploring nonnormative behaviors from a non-psychopathological perspective will help us to understand the evolution of sexual appetite as part of human sexual diversity and to prevent risky behaviors.

## Introduction

The World Health Organization (2020) indicates that sexuality includes sex, gender identity and role, sexual orientation, eroticism, pleasure, intimacy and reproduction, represented and expressed in thoughts, fantasies, desires, beliefs, attitudes, values, behaviors, practices, roles and relationships. In recent years there has been some uncertainty about the terms defining non-normative sexual behaviors, in other words, paraphilias and paraphilic disorders. Since the publication of DSM-III (American Psychiatric Association (APA), 1980), the focus on these behaviors has shifted from a psychoanalytic to an is atheoretical perspective. In the DSM-IV (American Psychiatric Association (APA), 1994) the distinction between paraphilia and paraphilic disorder was already present in the DSM-IV, as it established two sets of criteria. Criterion (A) defined paraphilia as “recurrent, intense sexually arousing fantasies, sexual urges, or behaviors generally involving (1) nonhuman objects, (2) the suffering or humiliation of oneself or one’s partner, or (3) children or other nonconsenting persons, that occur over a period of at least 6 months. For some individuals, paraphiliac fantasies or stimuli are essential for erotic arousal and are consistently integrated into sexual activity. In other cases, paraphiliac preferences manifest only episodically (e.g., during periods of stress), while at other times, the individual can function sexually without such fantasies or stimuli.” Criterion (B) specified that “the behavior, sexual urges, or fantasies cause clinically significant distress or impairment in social, occupational, or other important areas of functioning,” which, together with Criterion (A), constitutes the definition of a paraphilic disorder. The DSM-5 has not significantly altered these criteria, aside from the inclusion of “harm to others…” This perception was also influenced by the late conceptualization of sexology and sexuality research in the 19th century (Beccalossi et al., 2023) and, with it, the term paraphilia, which appeared in the 20th century to eliminate the concept of “sexual perversions.” However, authors such as Moser (2019) discuss how the DSM-5 conceptualises paraphilias and the implications of considering certain sexual behaviors as deviant. Băjenescu (2022) refers to sexual orientation as an internal and stable tendency to have psychological reactions of a sexual nature and desire for sexual relations with persons of a different sex or of the same sex. Sexual orientation has traditionally been classified as heterosexual, homosexual and bisexual, and from a binary perspective. Bogaert and Skorska (2020) refer to relatively stable sexual attraction to persons of the other sex (heterosexuality), to persons of the same sex (homosexuality) or to persons of both sexes (bisexuality). Although there are behaviors that could be called homosexual, for example, an erotic kiss between two men, there are authors who consider that their sexual orientation does not have to be homosexual or bisexual. To understand this, reference is made to Alfred C. Kinsey and his collaborators who between 1948 and 1953 published what is known as the Kinsey Report, which from its results, a scale was elaborated where the minimum score, 0, represented complete heterosexuality and the maximum score, 6, was homosexuality without more; on the other hand, if they obtained between 1 and 5, it was considered bisexuality, with 3 being the degree of complete bisexuality (Kinsey, 1948; Kinsey et al., 1953). In this way, sexual orientation can be seen as a spectrum. That is, it does not only take into account sexual orientation. This allows the authors to consider a person’s sexual behaviors and feelings, placing them on a continuum (Băjenescu, 2022). Over the years, the categories that encompass the term “sexual orientation” have become increasingly more complex and pragmatic (Watson et al., 2020; White et al., 2018), as the concept was previously limited due to various factors that influenced society, such as religion (Beltrán, 2012). Although in recent years there has been a trend towards a decrease in religiosity in Spain (Panadero et al., 2022), there are studies that still confirm that this religious influence is still present (Hone et al., 2020). Swaab (2007) states that sexual orientation can be influenced by a variety of factors, ranging from biological to social.

Besides sexual orientation, one of the other major factors determining sexual preferences and behavior is age (Browning et al., 2000; Herbenick et al., 2010). Most studies found significant changes in some individuals’ sexual behaviors not only over the course of their lives (Caltabiano et al., 2020: Chandra et al., 2013), but also between different generations (Twenge et al., 2015, 2017). Recently there has been a huge discussion about how to classify generations by giving them a label or name depending on the characteristics of the people who make them up. Studies such as those by Rainer and Rainer (2011) and Dimock (2019) state that the generation known as millennials is composed of people born between 1981 and 1996. Centennials, range from 1997 to 2005, considering only those over the age of majority (18 years). The classification of people according to age is done in order to be able to explain why we behave in a certain way from a general point of view.

Regarding the notion of ‘sexual appetite’, which represents a relevant aspect of our work, it will be conceptualised in its meaning of sexual desire. Such desire is constituted as the sum of the forces that drive us towards sexual behavior or distance us from it, oscillating its intensity in a dimension whose poles fluctuate between aversion and passion, evolving notably throughout life (Levine, 2003). Moreover, there may be gender discrepancies in the manifestation of desire, which may affect sexual and partner satisfaction (Mark and Murray, 2012).

The present study focusses on the most common non-normative sexual behaviors such as sadomasochism, which is one of the most popular paraphilias in general population, but is not as frequent as exhibitionism and voyeurism, for example (Bártová et al., 2021; Seto et al., 2021). This would be the combination of sexual sadism and sexual masochism, which according to the DSM-5 (American Psychiatric Association (APA), 2014) would be defined as the feeling of pleasure or sexual arousal when the person is shamed, beaten, tied up or subjected to some kind of abuse; and/or sexual arousal when any kind of suffering is inflicted on another person. Also, another very well-known paraphilic behavior is exhibitionism, in which sufferers experience sexual desire and pleasure by exposing their genitals or having sex in public while being observed (Seeman, 2020). Foot fetishism, bestiality, coprophilia and urophilia, although less common practices have also been included in the study, as they are still relevant for the classification of these behaviors (Krueger et al., 2017; Di Lorenzo et al., 2018). While it is true that some of these practices are more harmful to people’s health than others, they are all considered non-normative behaviors and have therefore been integrated into this study.

To explore the differences between millennials and centennials in appetite for non-normative sexual behaviors, the following hypotheses have been proposed.

H1: There will be differences in sexual orientation distribution between centennials and millennials.

In the DSM-I (American Psychiatric Association (APA), 1952), having a sexual orientation non-heterosexual was pathologised in the manual as “sexual deviations.” However, for clinical psychology, this term was replaced by “Egodystonic Homosexuality” in the publication of the DSM-III (American Psychiatric Association (APA), 1980), but was eventually deleted six years later in the review of that edition within the manual.

Considering that it was not until the 20th century that all issues related to sexuality began to be studied (Beccalossi et al., 2023), there have been statistical research that correlate sexual orientation according to age or the generation to which they belong. Based on statistics from different regions, people aged approximately 18–25 years are more likely to have sexual orientations other than heterosexual in both males and females (Copen et al., 2016; Gilmour, 2019). Some studies carried out in the Spanish population, claim that centennials, compared to millennials (Rainer and Rainer, 2011), have a higher percentage of people who are homosexual/bisexual or who are open to trying sex with someone of the same gender (Slebodnik, 2018). It is essential to highlight that in Spain there has been a legal evolution that protects gay, lesbian and bisexual people, for example, the approval of same-sex marriage. As a result, millennials and centenarians have lived through social, historical and political moments that have influenced their openness when it comes to communicating their non-normative sexual orientation. Despite this data, as the years go by, the figures are becoming more and more similar between the two generations.

In recent years, different research has been made into sexual orientation, in which it has been studied whether there are differences in sexual orientation according to different variables such as the role of hormones, evolutionary adaptation, genetics or cerebral and emotional differentiation (Soler, 2005). It has not been until more recently that the influence of age or the generation to which the subjects belong on their sexual orientation has been studied.

Following the same line, it has also been studied how age/generation would not only influence sexual orientation, in this case, on the opinions, attitudes and beliefs that people have towards homosexual and/or bisexual people (Costa et al., 2019; Ekstam, 2022).

H2: There will be differences in appetence for non-normative sexual behavior between centennials and millennials.

According to Cabello (2006), our sexual patterns have changed throughout history according to the political moment in which we find ourselves, since here in Spain, depending on the time period, we have been both in situations of sexual counter-education and in times when we have been able to talk freely about our sexuality.

Along the same lines, the ideological influence of religion in Spain has influenced people’s sex education and is therefore one of the justifications that show us the results of the studies to demonstrate that depending on the generation to which they belong, they will have certain preferences or others with respect to sexuality and thus to non-normative behaviors (Pérez, 2020).

This has led to greater sexual openness among young people over the years. One of the evidences is that in Spain, the average age of initiation of sexual relations with and without penetration has decreased to 16.5 years in 2018. In contrast, in 2010 a study was carried out which stated that the average age in Spain at that time was 17.9 years, which gives us an indication that the younger generation, centennials, is more sexually open than millennials (Díaz, 2010; Moreno et al., 2018).

There is currently an increase in sexual openness in the new generations due to the fact that patterns of sexual behavior are disappearing. In the past, girls were mainly looking for an affective-relational relationship and boys for relationships directly related to sexual pleasure, and were more sexually open than girls (Ballester and Gil, 2006; Browning et al., 2000). This sexual openness has also led to a 149 higher prevalence of risky behaviors today than in previous generations (Dhanoa et al., 2020). A dual pattern was thus established which, although it is more clearly observed in the affective than in the sexual sphere, seems to be changing (López et al., 2011).

However, according to DSM-5 (American Psychiatric Association (APA), 2014), there are 8 types of paraphilias which are sexual sadism, sexual masochism, foretturism, paedophilia, transvestism, fetishism and voyeurism. Exhibitionism, voyeurism and paedophilia are the most frequent paraphilias in men, while in women, masochism is the most common (Joyal and Carpentier, 2017). Sadism, sexual masochism and exhibitionism have been shown to be more likely to be practised by centennials than by millennials (Holvoet et al., 2017).

H3: There will be differences in preferences for non-normative sexual behaviors between millennials and centennials according to their sexual orientation.

When we talk about centennials, we are referring to one of the most recent/modern generations and that partly belongs to the 21st century. Thus, according to the study by Cañizo and Salinas (2010), it shows that there is a greater sexual permissiveness, in other words, being more open to having sexual relations or anything to do with sexuality, in young people than in adults.

It has also been shown that Spanish people belonging to this generation are more likely to have a homosexual or bisexual sexual orientation and there are researchers such as Cantillo (2013) who affirms, according to the results of their studies, that homosexual/bisexual people are more sexually open than heterosexuals.

Based on a study conducted among students at the Universidad del Atlántico, homosexual or bisexual individuals and/or couples have a less conventional and more sexually open sex life than heterosexual couples, therefore following the same line, they are more likely to practice non-normative behaviors (Cantillo, 2013).

Although it has been proven that homosexuals/Bisexuals are more likely to engage in this type of sexual behavior, this does not mean that heterosexuals follow a more traditional model, as some authors think, but rather that they are more likely to engage in other types of sexual behavior (Holvoet et al., 2017; Richters et al., 2008).

## Materials and methods

### Design and sampling

The study was conducted using a cross-sectional observational design with convenience sampling (Table 1).

**Table 1:** Sampling procedure datasheet.

Collection	07/04/2023 to 23/05/2023
Population	Centennials (1997–2005) and millennials (1981–1996) residents in Spain.
Population size	8,000,000 centennials 10,000,000 millennials
Sampling method	Convenience sampling
Survey type	Online
Confidence level	99%
Margin of error	10%
Sample size*	160 centennials 170 millennials

* The values are approximated base on the confidence level and the margin of error.

Although this type of design has some limitations, its use is indicated for the exploration of concepts that have not been widely studied and do not have validated measurement tools (Lefever et al., 2007; Nayak and Narayan, 2019), as is the case with non-normative sexual behavior.

### Procedure

Participants were invited to participate in the study through email and social media. The researchers also shared the information to participate through the dissemination channels provided by the XXX university in southern Spain (Andalusia). All participants received informed consent and the questionnaire (Annex A). The questionnaire was drafted *ad-hoc* and included three demographic items to describe the sample and the 3-point desire measurement for non-normative sexual behavior. Responses were collected via a link to the SurveyMonkey platform.

Participants had to be born between 1981 and 2005, reside in Spain when the survey was sent out and have a good understanding of Spanish. The records of participants who did not meet these criteria were removed from the study.

These responses did not contain any data that could reveal the identity of the participants and were accessible only to the study researcher in charge of data analysis.

### Measurement

Some previous studies have used validated scales for the assessment of paraphilic interest (Bártová et al., 2021; Joyal and Carpentier, 2017; Seto et al., 2021), but they are clinically oriented and determined by the DSM-5 diagnostic criteria. As there were no scales for the assessment of sexual desire for normative and non-normative sexual behaviors, a 3-point set of items (1 = Not appetizing; 2 = Indifferent; 3 = Appetizing). Although, it’s important to consider the limitations of a 3-point Likert scale, they can simplify responses and be effective positioning the participants in their response to indiscreet questions (Joshi et al., 2015). The scale used was drafted based on the behaviors most frequently referred to in the literature (Table 2).

**Table 2:** Literature used as reference for the design, drafting and selection of items.

Items	Authors
1. Having sex in a public place	Wylie (2015) and Yule et al. (2017)
2. Masturbating in a public place	
3. Being pressured to have sex or engage in any sexual conduct	Knight (2010), Zinik and Padilla (2016), and Agalaryan and Rouleau (2014)
4. Forcing someone to have sexual intercourse or perform sexual conduct	
5. Spanking or hitting someone during or prior to sexual intercourse	Labrecque et al. (2021), Balon (2014), and Seto et al. (2012)
6. Being whipped or beaten during or as a pre-sexual intercourse conduct	
7. Being tied up during or prior to sexual intercourse	Labrecque et al. (2021), Friedrich and Gerber (1994), and Cardoso (2022)
8. Tying someone up during or prior to sexual intercourse	
9. Practising choking (being grabbed by the neck) during sexual intercourse	
10. Practising choking (grabbing my partner’s neck) during sex sexual intercourse	
11. Watching someone naked	Normative sexual behaviors
12. Watching another person while masturbating or having sex	
13. Being watched while naked	
14. Being watched while masturbating or having sex	
15. The possibility or reality of having sex with an animal	Beetz (2004), Holoyda et al. (2018), and Joyal and Carpentier (2017)
16. Having sex with an inanimate object (food, dolls, etc.)	
17. Being aroused by fabrics or clothing	
18. Watching someone urinate or being urinated on	Milner et al. (2008) and Briken et al. (2016)
19. Urinating on your partner	
20. Defecating on your partner	
21. Watching someone defecate or being defecated on	
22. Wearing clothes of the opposite sex	Lawrence (2011), Agnew (2001), and Block (2015)
23. Seeing bare feet	
24. Being kissed on the feet	
25. Rubbing against someone without their consent in a public place	Di Lorenzo et al. (2018) and Balon (2016)

### Ethical considerations

This study was conducted in compliance with the Declaration of Helsinki of 2013 and the Organic Law 3/2018, 5th December, on the Protection of Personal Data and Guarantee of Digital Rights in Spain.

An informed consent text was sent to all participants together with the questionnaire. The participants were guaranteed confidentiality and anonymity.

### Data analysis

The frequencies and basic descriptive statistics of the variables in the questionnaire were analysed. The normality of the quantitative variables was verified using the Kolmogorov–Smirnov-Lilliefors test (Lilliefors, 1967). In addition, contingency tables were prepared and *χ*^2^ tests were performed between the generation and the sexual orientation in order to verify the existence of relationships between the crossed variables.

To contrast the differences in non-normative sexual behaviors, the Mann–Whitney U and Kruskall-Wallis tests were performed to test for differences in the range of values of numerical variables that do not fit the normal distribution (McKnight and Najab, 2010).

Data analysis and processing was performed with the software IBM Statistical Package for the Social Sciences (SPSS) v29. The statistical significance threshold was set at *p* < 0.05.

## Results

### Participants and overall responses

A sample of 332 young people from Spain was used. Of these young people, 159 were centennials (1997–2005), and 173 were millennials (1981–1996). Most of them identified themselves as woman (84.6%) and only 51 participants identified as man (15.4%). The mean age of the first group was 22.1 years (SD = 1.63), and the second group was 29.7 years (SD = 4.61). Regarding sexual orientation, 84.4% of millennials reported themselves as heterosexual (146), while 15.6% reported themselves as homosexual or bisexual (27). Among participants belonging to centennials, 73.6% would define themselves as heterosexual (117) and another 26.4% as homosexual or bisexual (42). The overall responses to the 3-point sexual desire instrument for the non-normative sexual behaviors are shown in Table 3.

**Table 3:** Overall responses to the sexual desire for the non-normative sexual behaviors.

Items		*n*
1. Having sex in a public place:	Not appetizing (1)	47
	Indifferent (2)	85
	Appetizing (3)	200
2. Masturbating in a public place:	Not appetizing (1)	156
	Indifferent (2)	111
	Appetizing (3)	65
3. Being pressured to have sex or engage in any sexual conduct:	Not appetizing (1)	175
	Indifferent (2)	44
	Appetizing (3)	113
4. Forcing someone to have sexual intercourse or perform sexual conduct:	Not appetizing (1)	187
	Indifferent (2)	69
	Appetizing (3)	76
5. Spanking or hitting someone during or prior to sexual intercourse:	Not appetizing (1)	74
	Indifferent (2)	112
	Appetizing (3)	146
6. Being whipped or beaten during or as a pre-sexual intercourse conduct:	Not appetizing (1)	71
	Indifferent (2)	66
	Appetizing (3)	195
7. Being tied up during or prior to sexual intercourse:	Not appetizing (1)	38
	Indifferent (2)	50
	Appetizing (3)	244
8. Tying someone up during or prior to sexual intercourse:	Not appetizing (1)	24
	Indifferent (2)	76
	Appetizing (3)	232
9. Practising choking (being grabbed by the neck) during sexual intercourse:	Not appetizing (1)	215
	Indifferent (2)	32
	Appetizing (3)	85
10. Practising choking (grabbing my partner’s neck) during sex sexual intercourse:	Not appetizing (1)	199
	Indifferent (2)	74
	Appetizing (3)	59
11. Watching someone naked:	Not appetizing (1)	13
	Indifferent (2)	87
	Appetizing (3)	232
12. Watching another person while masturbating or having sex:	Not appetizing (1)	26
	Indifferent (2)	69
	Appetizing (3)	237
13. Being watched while naked:	Not appetizing (1)	83
	Indifferent (2)	79
	Appetizing (3)	170
14. Being watched while masturbating or having sex:	Not appetizing (1)	88
	Indifferent (2)	68
	Appetizing (3)	176
15. The possibility or reality of having sex with an animal:	Not appetizing (1)	327
	Indifferent (2)	2
	Appetizing (3)	3
16. Having sex with an inanimate object (food, dolls, etc.):	Not appetizing (1)	168
	Indifferent (2)	103
	Appetizing (3)	61
17. Being aroused by fabrics or clothing:	Not appetizing (1)	68
	Indifferent (2)	206
	Appetizing (3)	58
18. Watching someone urinate or being urinated on:	Not appetizing (1)	286
	Indifferent (2)	29
	Appetizing (3)	17
19. Urinating on your partner:	Not appetizing (1)	289
	Indifferent (2)	28
	Appetizing (3)	15
20. Defecating on your partner:	Not appetizing (1)	332
	Indifferent (2)	0
	Appetizing (3)	0
21. Watching someone defecate or being defecated on:	Not appetizing (1)	329
	Indifferent (2)	1
	Appetizing (3)	2
22. Wearing clothes of the opposite sex:	Not appetizing (1)	28
	Indifferent (2)	210
	Appetizing (3)	94
23. Seeing bare feet:	Not appetizing (1)	97
	Indifferent (2)	212
	Appetizing (3)	23
24. Being kissed on the feet:	Not appetizing (1)	100
	Indifferent (2)	122
	Appetizing (3)	110
25. Rubbing against someone without their consent in a public place:	Not appetizing (1)	302
	Indifferent (2)	14
	Appetizing (3)	16

The frequency and the possible relationship between the two main classificatory variables, the sexual orientation and the generation to which each participant belonged were examined (Table 4).

**Table 4:** Frequency and percentage of participants by sexual orientation and generation.

		Generations				Total	
		Centennials		Millennials			
		*n*	%	*n*	%	*n*	%
Orientation	Heterosexual	117	73.6%	146	84.4%	263	79.2%
	Homosexual/Bisexual	42	26.4%	27	15.6%	69	20.8%
Total		159	100%	173	100%	332	100%

The results showed that the relationship between the two variables was significant (Continuity Correction = 5.241, *p* < 0.05; McNemar <0.001), so there were significant differences between them, and it was considered appropriate to explore these differences in the sexual appetite for non-normative behaviors.

As shown in Table 5, no differences were found between most non-normative sexual behaviors between generations. However, the hypothesis cannot be rejected entirely as there is a significant difference in non-normative behaviors related to the excitement of watching or being watched or to foot fetishism, with millennial participants being those with the highest mean rank in every significant comparison.

**Table 5:** Differences in appetite for non-normative sexual behavior between centennials and millennials.

		*n*	Mean rank	U Mann–Whitney	*p*
1. Having sex in a public place	Centennials (1997–2005)	159	160.70	12831.000	0.226
	Millennials (1981–1996)	173	171.83		
2. Masturbating in a public place	Centennials (1997–2005)	159	164.79	13482.000	0.736
	Millennials (1981–1996)	173	168.07		
3. Being pressured to have sex or engage in any sexual conduct	Centennials (1997–2005)	159	165.62	13614.000	0.859
	Millennials (1981–1996)	173	167.31		
4. Forcing someone to have sexual intercourse or perform sexual conduct	Centennials (1997–2005)	159	161.83	13011.000	0.342
	Millennials (1981–1996)	173	170.79		
5. Spanking or hitting someone during or prior to sexual intercourse	Centennials (1997–2005)	159	165.20	13546.500	0.799
	Millennials (1981–1996)	173	167.70		
6. Being whipped or beaten during or as a pre-sexual intercourse conduct	Centennials (1997–2005)	159	173.69	12610.000	0.138
	Millennials (1981–1996)	173	159.89		
7. Being tied up during or prior to sexual intercourse	Centennials (1997–2005)	159	169.37	13297.500	0.500
	Millennials (1981–1996)	173	163.86		
8. Tying someone up during or prior to sexual intercourse	Centennials (1997–2005)	159	165.27	13557.500	0.780
	Millennials (1981–1996)	173	167.63		
9. Practising choking (being grabbed by the neck) during sexual intercourse	Centennials (1997–2005)	159	175.33	12349.500	0.057
	Millennials (1981–1996)	173	158.38		
10. Practising choking (grabbing my partner’s neck) during sex sexual intercourse	Centennials (1997–2005)	159	170.92	13050.000	0.358
	Millennials (1981–1996)	173	162.43		
11. Watching someone naked	Centennials (1997–2005)	159	144.20	10207.500	0.000
	Millennials (1981–1996)	173	187.00		
12. Watching another person while masturbating or having sex	Centennials (1997–2005)	159	150.59	11223.500	0.000
	Millennials (1981–1996)	173	181.12		
13. Being watched while naked	Centennials (1997–2005)	159	155.94	12075.000	0.036
	Millennials (1981–1996)	173	176.20		
14. Being watched while masturbating or having sex	Centennials (1997–2005)	159	152.94	11597.500	0.007
	Millennials (1981–1996)	173	178.96		
15. The possibility or reality of having sex with an animal	Centennials (1997–2005)	159	167.14	13652.500	0.584
	Millennials (1981–1996)	173	165.92		
16. Having sex with an inanimate object (food, dolls, etc.)	Centennials (1997–2005)	159	157.56	12332.500	0.075
	Millennials (1981–1996)	173	174.71		
17. Being aroused by fabrics or clothing	Centennials (1997–2005)	159	162.07	13048.500	0.351
	Millennials (1981–1996)	173	170.58		
18. Watching someone urinate or being urinated on	Centennials (1997–2005)	159	165.31	13565.000	0.719
	Millennials (1981–1996)	173	167.59		
19. Urinating on your partner	Centennials (1997–2005)	159	163.75	13317.000	0.391
	Millennials (1981–1996)	173	169.02		
20. Defecating on your partner	Centennials (1997–2005)	159	166.50	13753.500	1.000
	Millennials (1981–1996)	173	166.50		
21. Watching someone defecate or being defecated on	Centennials (1997–2005)	159	166.05	13681.500	0.615
	Millennials (1981–1996)	173	166.92		
22. Wearing clothes of the opposite sex	Centennials (1997–2005)	159	160.31	12768.500	0.185
	Millennials (1981–1996)	173	172.19		
23. Seeing bare feet	Centennials (1997–2005)	159	149.40	11034.500	0.000
	Millennials (1981–1996)	173	182.22		
24. Being kissed on the feet	Centennials (1997–2005)	159	150.95	11281.500	0.003
	Millennials (1981–1996)	173	180.79		
25. Rubbing against someone without their consent in a public place	Centennials (1997–2005)	159	166.26	13715.500	0.930
	Millennials (1981–1996)	173	166.72		

Considering sexual orientation in the differences between generations, a higher number of between-group differences for non-normative behaviors were found. These were added to those related to watching/being watched and seeing or kissing feet, choking behaviors, the possibility or unreality of having sex with an animal, and behaviors related to urinating or being urinated (Table 6).

**Table 6:** Differences in appetite for non-normative sexual behavior between centennials and millennials among groups according to generation and sexual orientation.

		*n*	Mean rank	Kruskal-Wallis
1. Practising choking (being grabbed by the neck) during sexual intercourse	Centennials/Heterosexual	117	168.33	10.868
	Centennials/Bi-Homosexual	42	194.82	
	Millennial/Heterosexual	146	153.14	
	Millennial/Bi-Homosexual	27	186.72	
2. Watching someone naked	Centennials/Heterosexual	117	137.76	29.015
	Centennials/Bi-Homosexual	42	162.14	
	Millennial/Heterosexual	146	188.10	
	Millennial/Bi-Homosexual	27	181.06	
3. Watching another person while masturbating or having sex	Centennials/Heterosexual	117	137.79	27.355
	Centennials/Bi-Homosexual	42	186.24	
	Millennial/Heterosexual	146	184.08	
	Millennial/Bi-Homosexual	27	165.15	
4. Being watched while masturbating or having sex	Centennials/ Heterosexual	117	144.18	11.967
	Centennials/Bi-Homosexual	42	177.36	
	Millennial/Heterosexual	146	178.10	
	Millennial/Bi-Homosexual	27	183.61	
5. The possibility or reality of having sex with an animal	Centennials/Heterosexual	117	164.00	11.206
	Centennials/Bi-Homosexual	42	175.87	
	Millennial/Heterosexual	146	166.27	
	Millennial/Bi-Homosexual	27	164.00	
6. Watching someone urinate or being urinated on	Centennials/Heterosexual	117	160.05	9.929
	Centennials/Bi-Homosexual	42	179.99	
	Millennial/Heterosexual	146	162.94	
	Millennial/Bi-Homosexual	27	192.72	
7. Urinating on your partner	Centennials/Heterosexual	117	157.56	17.158
	Centennials/Bi-Homosexual	42	181.01	
	Millennial/Heterosexual	146	162.96	
	Millennial/Bi-Homosexual	27	201.81	
8. Seeing bare feet	Centennials/Heterosexual	117	141.49	17.810
	Centennials/ Bi-Homosexual	42	171.43	
	Millennial/Heterosexual	146	181.66	
	Millennial/Bi-Homosexual	27	185.20	
9. Being kissed on the feet	Centennials/Heterosexual	117	146.61	11.078
	Centennials/Bi-Homosexual	42	163.05	
	Millennial/Heterosexual	146	177.79	
	Millennial/Bi-Homosexual	27	196.98	

Only variables that showed significant differences (*p* < 0.05) and were significance with Monte Carlo resampling (with a confidence interval of 99% and based on 10,000 sampled tables with starting seed 2,000,000) are shown in this table.

## Discussion

Sexuality and related behaviors are an essential subject of interest in order to understand and prevent possible risky behaviors and reduce misinformation and stigma, especially among young people (Goldfarb and Lieberman, 2021). Prior studies have already addressed generational differences in appeal of sexual practices beyond the Western cultural hetero-normative framework (Ben Hagai et al., 2022; Campbell, 2022). However, the lack of studies approaching non-normative sexual behaviors in these aged groups, is partially due to the psychopathological conception which relates these behaviors directly to distress and the existence of a risk to the health of the person or their environment (Di Lorenzo et al., 2018), but also because of the disparity in categorising these behaviors (Joyal, 2021; Masiran, 2018). The current study considers an approach in this line, away from the psychopathological component of the behaviors and comparing the appetite for these behaviors without considering it necessary for there to be a negative assessment of sexual preference. We have focused on interest rather than behavior, as some studies have reported one can predict the other (Joyal et al., 2021). While it is true that some sexual behaviors have a psychopathological component, there are other behaviors and desires that could be part of healthy sexual diversity and are widely prevalent in the general population (Joyal and Carpentier, 2021). In order to discuss the results found, this section will be divided in order to respond to the hypotheses suggested.

Despite of the small sample size (*n* = 332), a significant relationship was found between the sexual orientation of the participants and their generation. This is concordant with what is presented in a large number of studies with wider samples (Twenge et al., 2015, 2017), which help us to support the representativeness of the sample for the other hypotheses. The majority of millennials and centennials claim to be mainly attracted by people with the opposite sex (approximately 79 and 69% respectively), according to a survey performed in the UK (Ipsos MORI, 2020). This paper also reveals that 18% of millennials are attracted to the same sex or consider themselves bisexual, and in the case of centennials this percentage rises to 26%. The Spanish Youth Institute’s annual youth survey, asked about the sexual orientation of young people between the ages of 15 and 29 years old. A total of 77.5% identified themselves as heterosexual and 16.6% identified either as homosexual or bisexual [Instituto de la Juventud (INJUVE), 2020]. Both studies mention the globalisation, the decrease in social stereotypes and equality policies as possible factors involved in the sexual liberation of centennials. These statistics are similar to those found in the study sample. In this case, the differences are also greater among millennials (73.6% heterosexual, 26.4% homosexual or bisexual) than among centennials (84.4% heterosexual, 15.6% homosexual or bisexual), with the overall result being somewhere in midpoint (79.2% heterosexual, 20.8% homosexual or bisexual). According to these results, the sample of the present study can be considered representative in relation to the sexual orientation of the participants, and the following hypotheses can be taken into account with this consideration in mind.

As can be seen in the results section, this study did not find a statistically significant difference, according to the results obtained, between the two generations in most of the non-normative sexual behaviors explored, which is not in line with the conclusions reached by López et al. (2011). Among the reasons which may argue these differences, there is the historical timing in which each study has been carried out. Even though the results of López et al. (2011) were found just over a decade ago, it is probable that the differences between generations at that moment in time were more accentuated than they are nowadays.

Nevertheless, there were significant differences between the appetence of both generations for behaviors related to voyeurism and exhibitionism (items 11, 12, 13 and 14) and behaviors related to foot fetishism (items 23 and 24). Within all of these behaviors, appetence marks were higher in the millennial group than in the centennials. There is no consistent evidence in the literature to justify these differences. However, it is probably due to the differences in digitisation between the two generations (Quincy and Manduza, 2021). Millennials dealt with the digitisation of society during adolescence and young adulthood, while centennials were born into a fully digitised world with access to the internet from an earlier age (Kaviani and Nelson, 2021). Premature exposure to stimuli that could be perceived as sexual may have affected centennials’ capacity for excitation and appetite for images of nudity or sexual scenes (Carvalho, 2021). This added to the fact of living in a more open society for the exploration of sexuality and the decreasing age at which the first sexual relations took place (Neff, 2020) can also explain the reduced desire to be seen undressed or to be observed having sex. Early exposure to pornography may also explain differences in sexual appetite to images of nudity or having sex (Massey et al., 2021), particularly with centennials having greater exposure to pornography at a younger age than millennials, who may be more sensitive to such content due the lack of early exposure to it (Ningrum and Kusbaryanto, 2021). In addition to the consumption of pornography, the increased use of mobile devices among teenagers has facilitated the development of sexual behaviors aimed at sharing nude images or videos or engaging in behaviors related to erotic self-stimulation such as sexting, which could contribute to the desensitization of millennials to seeing other people naked or engaging in sexual acts (Crofts et al., 2016; Raine et al., 2020). Another possibility is that the appetite for these paraphilic interests/behaviors may appear later in life (Chandra et al., 2013). Regarding the millennials’ increased appetite for foot fetish-related behavior (items 23 and 24), it could be explained by the rise of extreme and atypical content included in sexually explicit internet material (SEIM), especially content related to fetishism and other dominance/submission behaviors (The Economist, 2015). Such content can serve as models or scripts for sexual behavior among young people (Frith and Kitzinger, 2001). Thus, repeated exposure to pornography can give rise to sexual scripts where certain sexual behaviors, sexual roles, gender stereotypes, and attitudes are normalized and promoted (Ryan, 2011).

Regarding the third hypothesis, which concerns the differences in the appetite for more varied non-normative sexual behaviors in centennial participants compared to millennials, taking into account the variable of sexual orientation, the influence of sexual orientation on the appetite for different non-normative behaviors is confirmed. The non-normative behaviors that showed significant differences were the same as those in the second hypothesis (items 11, 12, 13, 14, 23, and 24), along with the appetite for practicing asphyxia (item 10), the desire or reality of having sex with animals (item 15), and seeing someone urinate on another person or urinating yourself on another person (items 18 and 19).

On the one hand, these results could be in line with those found by Cañizo and Salinas (2010), as there seems to be a greater appetite for non-normative sexual behaviors in younger people. Additionally, these findings support what Hunt et al. (2019) pointed out, who found a greater appetite for a wider variety of non-normative sexual behaviors in people with a homosexual or bisexual sexual orientation.

The appetite for engaging in asphyxia was found to be greater among homosexual/bisexual groups in both generations. This increased appetite may be due to the high prevalence of sexual aggression-related behaviors, including asphyxia, in gay pornography (Fritz and Bowling, 2022). Consumption of this type of content can lead to learning and an increased likelihood of replicating these behaviors in sexual practice. However, this type of content is more commonly found in pornography aimed at heterosexual audiences, in which aggression is typically directed towards women (Carrotte et al., 2020). Consumption of this type of content has been shown to influence the sexual behaviors of young people (Herbenick et al., 2022), with women and transgender/non-binary individuals being more likely to have been strangled than men. Further research is needed to understand the relationship between sexual orientation and the appetite for engaging in asphyxia in order to draw more comprehensive conclusions regarding the results of the present study.

Regarding the desire to engage in behaviors related to bestiality, it is centennials with a homosexual/bisexual orientation scored higher compared to other groups. These results may be related to the furry phenomenon, known as the sexual and affective attraction to anthropomorphic animals, cartoon animals, or people dressed as animals. Hsu and Bailey (2019) surveyed 334 men identified as furries to ask them questions about their sexual orientation, sexual motivation, and sexual interests. A large majority of our sample identified as non-heterosexual (84%) and reported some degree of sexual motivation to be a furry (99%). Although this phenomenon appears to have non-sexual motivations that have a greater impact on identification with the group members of this phenomenon (Brooks et al., 2022).

Regarding behaviors traditionally conceptualized within urophilia, such as watching someone urinate or being urinated on or urinating on a partner, homosexual/bisexual millennials were the group that showed the greatest appetite for these behaviors. If access to extreme, abnormal, or domination/submission-based pornography is becoming more accessible (The Economist, 2015), this may explain a greater desensitization of young people to such practices. According to a study by Vandenbosch (2015), adolescents are more exposed to sexually explicit internet material (SEIM) related to affectionate themes, while young adults are more often exposed to SEIM related to domination themes. Consequently, millennials appear to be more likely to be exposed to pornography with themes of domination and violence than centennials. This greater exposure to domination content could explain the greater appetite of millennials for behaviors related to foot fetishism and urophilia, although the relationship of the latter to sexual orientation has not yet been thoroughly explored.

In summary, no differences were found in the inclination to engage in most of the non-normative sexual behaviors studied between generations. However, it seems that sexual orientation is a variable that can help better assess these differences. These results can be very revealing about contemporary Spanish society, where understanding these behaviors from a perspective that moves away from the psychopathological approach can help design better education programs on sexual diversity without the usual restrictions based on prejudices and taboos (Pomeroy, 2017). In any case, further research with larger samples is needed to confirm or refute the findings of the present study.

However, we do conclude that there is a need for research that explores how to improve sexuality education programmes in Spain, from a holistic perspective with sexual and gender diversity, in order to increase the levels of physical, mental and sexual health of society as a whole.

## Data Availability

The raw data supporting the conclusions of this article will be made available by the authors, without undue reservation.
