# Supplementary material for: Sexual desire for non-normative sexual behaviors: differences between centennials and millennials considering sexual orientation
Source: Front Sociol. 2024 Dec 19;9:1509111. doi: 10.3389/fsoc.2024.1509111 (PMC11694149; doi:10.3389/fsoc.2024.1509111)
Supplement: Supplementary file 1 [file Table_1.pdf]

Appendix A

Age:

Sexual orientation:

Non-normative sexual behaviors

You will find some statements about sexual behavior. Read each statement carefully and indicate if they are Not appetizing, indifferent or appetizing for you.

(1 = Not appetizing; 2 = Indifferent; 3 = Appetizing).

Please answer as honestly as possible, in a way that shows how you really are, not how you would like to be or how you think you should. Don't spend too much time thinking about your answers. The first answer that pops into your head is what is needed.

	1	2	3
1. Having sex in a public place			
2. Masturbating in a public place			
3. Being pressured to have sex or engage in any sexual conduct			
4. Forcing someone to have sexual intercourse or perform sexual conduct			
5. Spanking or hitting someone during or prior to sexual intercourse			
6. Being whipped or beaten during or as a pre-sexual intercourse conduct			
7. Being tied up during or prior to sexual intercourse			
8. Tying someone up during or prior to sexual intercourse			
9. Practising choking (being grabbed by the neck) during sexual intercourse			
10. Practising choking (grabbing my partner's neck) during sex sexual intercourse			
11. Watching someone naked			
12. Watching another person while masturbating or having sex			

13. Being watched while naked
14. Being watched while masturbating or having sex
15. The possibility or reality of having sex with an animal
16. Having sex with an inanimate object (food, dolls, etc.)
17. Being aroused by fabrics or clothing
18. Watching someone urinate or being urinated on
19. Urinating on your partner
20. Defecating on your partner
21. Watching someone defecate or being defecated on
22. Wearing clothes of the opposite sex
23. Seeing bare feet
24. Being kissed on the feet
25. Rubbing against someone without their consent in a public place
